# Association of healthy lifestyle factors with the risk of hypertension, dyslipidemia, and their comorbidity in Korea: results from the Korea National Health and Nutrition Examination Survey 2019-2021

**DOI:** 10.4178/epih.e2024049

**Published:** 2024-05-01

**Authors:** Ji-Sook Kong, Mi Kyung Kim

**Affiliations:** 1 Department of Preventive Medicine, Hanyang University College of Medicine, Seoul, Korea; 2 Institute for Health and Society, Hanyang University, Seoul, Korea; 3 Department of Epidemiology and Health Statistics, Graduate School of Public Health, Hanyang University, Seoul, Korea

**Keywords:** Hypertension, Dyslipidemia, Comorbidity, Healthy lifestyle factors, Body mass index, Demographic factors

## Abstract

**OBJECTIVES:**

We investigated the association of individual healthy lifestyle factors (HLFs) and their combined healthy lifestyle score (HLS) with hypertension and/or dyslipidemia.

**METHODS:**

We analyzed data from 10,693 adults aged ≥19 from the 2019 to 2021 Korea National Health and Nutrition Examination Survey. HLS was evaluated based on smoking status, alcohol consumption, body mass index (BMI), diet, and physical activity. Using logistic regression models, we estimated odds ratios (ORs) with 95% confidence intervals (CIs) to evaluate the associations of HLFs and HLS with hypertension, dyslipidemia, and their comorbidity.

**RESULTS:**

The prevalence of hypertension alone, dyslipidemia alone, and their comorbidity was 8.7%, 24.6%, and 15.0%, respectively. Multivariable models showed an inverse association of hypertension (OR, 0.37; 95% CI, 0.30 to 0.46) and dyslipidemia (OR, 0.36; 95% CI, 0.32 to 0.41) with healthy BMI. Hypertension was inversely associated with healthy alcohol consumption (OR, 0.46; 95% CI, 0.35 to 0.61) and diet (OR, 0.79; 95% CI, 0.63 to 0.99), whereas dyslipidemia was inversely associated with non-smoking (OR, 0.51; 95% CI, 0.43 to 0.60). Physical activity was inversely associated with their comorbidity (OR, 0.69; 95% CI, 0.56 to 0.85). Adherence to HLS was associated with significantly lower odds of hypertension (81%), dyslipidemia (66%), and their conditions (89%) (all p_trend_<0.001). Stratified analyses consistently showed inverse associations between HLS and hypertension and/or dyslipidemia independently of demographic factors (p_interactions_>0.05).

**CONCLUSIONS:**

HLFs were associated with lower risk for hypertension and/or dyslipidemia. Obesity may contribute significantly to the risk of these conditions, while relevant HLFs for individual chronic diseases may vary significantly.

## GRAPHICAL ABSTRACT

[Fig f2-epih-46-e2024049]

## Key Message

Individual components of healthy lifestyle factors (HLFs) were inversely associated with the risk of comorbid hypertension and dyslipidemia, as well as with each condition. Among the various HLFs, BMI status was identified as a significant factor, while relevant HLFs for individual chronic diseases may vary significantly. Additionally, clear dose-response associations were observed, indicating that adherence to more HLFs was significantly associated with decreasing odds of hypertension, dyslipidemia, and their comorbidity.

## INTRODUCTION

Hypertension and dyslipidemia are globally widespread noncommunicable diseases that impose a substantial burden on public health because they are pivotal risk factors for cardiovascular disease (CVD) and mortality [1-3]. Despite efforts to increase awareness and treatment in Korea [4], the prevalence of hypertension has remained relatively stable, whereas the absolute number of individuals with hypertension has continued to increase steadily [5]. Additionally, there has been a reported increase in the incidence of hypercholesterolemia [6]. Notably, hypertension and dyslipidemia often coexist, either independently or synergistically, significantly contributing to CVD [7-9]. Individuals with both dyslipidemia and hypertension face substantially higher risks of CVD events and mortality than those with only one of these conditions [7-9]. This highlights the critical importance of addressing these comorbidities together in primary prevention strategies to reduce the overall disease burden.

Several meta-analyses of prospective studies have shown that adopting a combination of healthy lifestyle factors (HLFs) or indices can significantly reduce the risk of CVD and all-cause mortality [10,11]. These studies considered cigarette smoking, alcohol consumption, physical activity (PA), diet, and body mass index (BMI) as combined lifestyle factors and indices. However, in the context of hypertension and dyslipidemia development, although increasing evidence highlights the importance of HLFs [1,12,13], only a few studies have specifically investigated the association between individual HLFs and dyslipidemia, as well as their comorbidity [14]. Moreover, it is crucial to acknowledge that these HLFs do not function in isolation; rather, they are part of an interconnected web of factors in real-life contexts [11-14]. Therefore, it is necessary to investigate the combined effects of HLFs on these outcomes. Additionally, despite the recognition of various established risk factors such as age, socioeconomic status, and family history (FH) for chronic conditions including hypertension and dyslipidemia [1], there is still a lack of data on the interaction between HLFs and these covariates [12,14-16].

Therefore, we aimed to investigate the associations between individual HLFs and their combined healthy lifestyle score (HLS) and the risk of hypertension and/or dyslipidemia.

## MATERIALS AND METHODS

### Study population

The Korea National Health and Nutrition Examination Survey (KNHANES) is conducted by the Korea Disease Control and Prevention Agency (KDCA). We analyzed data from the KNHANES collected between 2019 and 2021. This survey sampled participants from Korean households using a stratified multistage probability sampling design to ensure a population-based representation. Data collection was carried out by trained investigators who adhered to a standardized protocol. Detailed information about the KNHANES can be found in a previous publication [17] and on the official website (https://knhanes.cdc.go.kr/).

A total of 22,559 participants completed the KNHANES 2019-2021 survey. We excluded participants for the following reasons: age under 19 years or over 80 years (n=4,982); missing information on hypertension or dyslipidemia; non-fasting status (n=1,827); and/or a history of any cancer, current pregnancy, or breastfeeding (n=847). Additionally, participants with incomplete data on any of the following variables were also excluded (n=4,210): age, education level, household income status, marital status, energy intake, BMI, smoking status, drinking status, PA, healthy diet, prior diagnoses of hypertension and/or dyslipidemia, and FH of hypertension and dyslipidemia. After these exclusions, 10,693 participants (male: 4,663, female: 6,030) remained for the final analysis (Supplementary Material 1).

### Study outcomes

#### Measurement of BP and definition of hypertension

Blood pressure (BP) was measured with a mercury sphygmomanometer (Baumanometer Wall Unit 33(0850); W.A.Baum, New York, NY, USA) in 2019, a non-mercury auscultatory BP device (Greenlight 300) in 2020, and a non-mercury oscillometric BP device (Microlife WatchBP Office AFIB; Microlife AG, Widnau, Switzerland) in 2021. Using the average of the second and third measurements of systolic BP (SBP) and diastolic BP (DBP), hypertension was defined as (1) SBP ≥ 140 mmHg and/or DBP ≥ 90 mmHg [18], or (2) self-reported use of anti-hypertensive medication for the purpose of BP control.

#### Blood sampling and definition of dyslipidemia

Blood samples were collected from the antecubital vein in the morning after an overnight fast. Lipid profiles, including total cholesterol (TC), high-density lipoprotein cholesterol (HDL-C), triglycerides (TG), and low-density lipoprotein cholesterol (LDL-C), were measured enzymatically using a Hitachi Labospect 008AS Analyzer (Hitachi, Tokyo, Japan). When direct methods for measuring LDL-C levels were available (13.1% of participants), they were used. If the TG level was below 400 mg/dL and direct measurement was not possible, LDL-C levels were calculated indirectly using the Friedewald formula [LDL-C = TC−HDL-C−(TG/5)] [19]. Dyslipidemia was defined according to the criteria set forth by the Expert Panel on Detection, Evaluation, and Treatment of High Blood Cholesterol in Adults from the National Cholesterol Education Program [20] and the 2022 Korean guidelines for the diagnosis of dyslipidemia [21], dyslipidemia was defined as meeting any of the following criteria in individuals who had fasting periods of 9 hours or more: (1) hypercholesterolemia, indicated by TC level of ≥ 240 mg/dL; (2) hypertriglyceridemia, indicated by a TG level of ≥ 200 mg/dL; (3) hyper-LDL-cholesterolemia, indicated by an LDL-C level of ≥ 160 mg/dL; (4) hypo-HDL-cholesterolemia, indicated by an HDL-C level of < 40 mg/dL; (5) having received a medical diagnosis of dyslipidemia by a physician; and/or (6) undergoing treatment with anti-dyslipidemia medication to control TC.

### Study exposures

#### Classification of HLFs

Five lifestyle factors (i.e., smoking, alcohol consumption, BMI, diet, and PA) were used to construct the HLS [8,11,22,23]. Health-related behaviors, including smoking status, alcohol consumption, fruit and vegetable intake, and PA, were assessed using a structured questionnaire. Anthropometric measurements of height and weight were obtained to calculate BMI, which was determined by dividing the weight (kg) by the square of the height (m^2^). These factors were categorized as either low risk (aligned with a healthy lifestyle) or high risk (not aligned with a healthy lifestyle) in relation to the risk of hypertension or dyslipidemia (Table 1). Nonsmokers were defined as individuals who had never smoked, were former smokers, or had smoked fewer than 100 cigarettes in their lifetime. Healthy alcohol consumption was defined as ≤ 2 drink/day for male and ≤ 1 drink/day for female, according to the Dietary Guidelines in the United States [24]. A healthy BMI was defined as a BMI < 25 kg/m^2^ [25]. A healthy diet was defined as consuming a median amount or more of fruits and vegetables per day (3 times) [26]. Healthy PA was defined as meeting the World Health Organization (WHO) guidelines, which include engaging in < 150 minutes of moderate-intensity aerobic PA per week, at least 75-150 minutes of vigorous-intensity aerobic PA, or an equivalent combination of moderate-intensity and vigorous-intensity PA throughout the week [27], using the Global Physical Activity Questionnaire [28].

#### Calculation of the HLS

To assess the combined influence of HLFs, participants were assigned a score of 1 for low risk and 0 for high risk. The HLS was calculated by summing the scores assigned to the 5 factors mentioned above. HLS values ranged from 0 to 5, indicating the degree of adherence to a healthy lifestyle. Due to the small percentage of participants scoring between 0 and 1, the HLS was further categorized into groups: 0 to 1, 2, 3, 4, and 5.

#### Covariates

Data on demographic and health-related variables were gathered through face-to-face interviews. Demographic factors such as sex, age, education level, household income, and marital status were evaluated using structured questionnaires. Education was divided into 4 categories: elementary school graduate or below, middle school graduate, high school graduate, and college graduate or higher. Household income was divided into 4 quartiles: low, low-middle, middle-high, and high. Health-related variables included an FH of parental hypertension/dyslipidemia and previous physician diagnoses of hypertension and/or dyslipidemia, to account for the potential influence of reverse causality.

### Statistical analysis

To account for the complex sampling design, primary sampling units, strata, clusters, and weights were applied to all analyses (PROC SURVEY) to represent the Korean population. Data are presented as mean± standard error for continuous variables and as weighted percentages for categorical variables. Logistic and multinomial logistic regression models were utilized to estimate odds ratios (ORs) with 95% confidence intervals (95% CIs), adjusting for confounders. We used three models: model 1 adjusted for age and sex; model 2 further adjusted for age, sex, education level, household income status, marital status, energy intake, prior physician diagnoses of hypertension and/or dyslipidemia, and FH of hypertension and/or dyslipidemia; and model 3 (i.e., the final model), which included adjustments for all covariates in model 2 plus additional lifestyle factors (the correlation matrix among these lifestyle factors is available in Supplementary Material 2). This analysis aimed to assess the association between individual factors and HLS, and the risk of hypertension, dyslipidemia, and comorbidity. Furthermore, we explored the association between HLS and outcomes by stratifying participants into different groups based on age (5,253 participants aged < 50 and 5,440 participants aged 50 or older), household income (4,127 participants with lower income and 6,566 participants with higher income), BMI groups (6,874 participants with a BMI < 25 kg/m^2^ and 3,819 participants with a BMI of ≥ 25 kg/m^2^), and FH of parental hypertension or dyslipidemia (4,565 participants with FH and 6,128 participants without FH).

To test the robustness of the associations between individual exposure variables and our outcomes, we conducted several sensitivity analyses. These analyses explored the associations using different approaches: (1) substituting BMI with waist circumference (WC) and waist-to-height ratio (WHtR); (2) replacing the original PA variable with variables for walking time, muscle exercise, and seating time [27]; (3) using a criterion of consuming fruits and/or vegetables 5 times and 4 times per day, and considering individual fruits and vegetables, instead of the original benchmark of 3 times/day as median intakes [26]; (4) evaluating sodium-to-potassium ratio or sodium density (mg/1,000 kcal) as alternatives to the original dietary factor [29,30] (Supplementary Material 3). Additionally, to examine interactions with other established risk factors such as age, household income, BMI, and FH of hypertension and dyslipidemia, we analyzed the association between individual HLFs and outcomes. This was done by stratifying participants into groups based on sex (4,663 male and 6,030 female), age, household income, BMI, presence or absence of prior physician diagnoses, and FH of parental hypertension or dyslipidemia (Supplementary Materials 4-9). All statistical analyses were performed using SAS version 9.4 (SAS Institute Inc., Cary, NC, USA). A p-value of < 0.05 was considered statistically significant.

### Ethics statement

The KNHANES 2019-2021 received approval from the Institutional Review Board of the KDCA, in accordance with the principles outlined in the Declaration of Helsinki. The approval numbers are 2019-2021, 2018-01-03-C-A, 2018-01-03-2C-A, and 2018-01-03-5C-A. Written informed consent was obtained from all participants.

## RESULTS

The characteristics of the 10,693 participants are shown in Table 2. Their average age was 42.5± 0.3 years. The prevalence of hypertension and dyslipidemia was 23.7% and 39.6%, respectively. Among the 5 HLFs, a larger proportion of participants were nonsmokers and engaged in healthy alcohol consumption, whereas adherence to factors such as PA and a healthy diet was relatively low. Participants with higher HLS tended to be older and had a higher proportion of college graduates or individuals with further education, higher income, and a higher rate of marriage. Additionally, individuals with higher HLS exhibited lower values of obesity indices, such as BMI and WC, and showed favorable profiles of BP and lipid parameters, including cholesterol, TG, LDL-C, and HDL-C. Moreover, those with higher HLS had a lower prevalence of hypertension and dyslipidemia.

Table 3 presents the associations between individual HLFs and the risk of hypertension and/or dyslipidemia. In the final model, an inverse association was found between hypertension and factors such as healthy BMI, healthy alcohol consumption, and healthy fruit and vegetable intake. Conversely, dyslipidemia was inversely associated with healthy BMI and non-smoking status. However, no significant association was found between PA and any of the outcomes. Despite this, significant inverse associations were observed between comorbidity of hypertension and dyslipidemia and all factors, including healthy BMI, non-smoking status, healthy alcohol consumption, and healthy PA, with the exception of fruit and vegetable intake.

Table 4 presents the association between HLS and the risks of hypertension and dyslipidemia. With increasing HLS, there was a clear trend of reduced odds for the risks of hypertension and dyslipidemia individually, as well as for their comorbidity. These findings remained significant in both the age-adjusted and sex-adjusted model and the multivariable model, highlighting the substantial impact of HLFs beyond these demographic factors.

**Figure 1.**
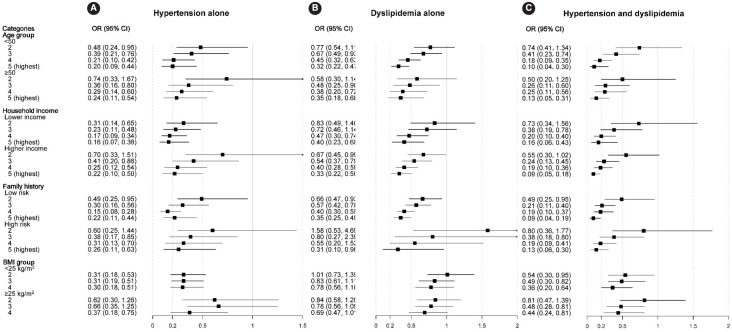
Association between healthy lifestyle score and risk of hypertension and/or dyslipidemia by covariates. The multivariable model was adjusted for age (years), sex, education status (≥12 years of school), household income status (≥3 times/wk and ≥30 min/session), marital status, energy intake (kcal/day), diagnosis of hypertension and/or dyslipidemia by physician, and family history of hypertension and/or dyslipidemia (except for analysis stratified by family history). OR, odds ratio; CI, confidence interval; BMI, body mass index.

Figure 1 illustrates the association between HLS and the outcomes by stratifying the participants into groups based on other established risk factors such as age, household income, FH of these conditions, and BMI, all of which are significant risk factors for CVD. Although there was a significant interaction between HLS and comorbidity across age groups (p<0.05), our analyses showed that these factors did not result in substantial differences in the overall outcomes.

We conducted multiple sensitivity analyses to assess the robustness of our findings. For obesity, we employed alternatives to the commonly used BMI measurements, such as WC and WHtR. In evaluating the PA factor, we explored alternatives by examining variables like walking duration, muscle strength, and sedentary time. In terms of dietary factors, we investigated the consumption of fruits and/or vegetables 5 times and 4 times per day, as well as individual types of fruits and vegetables, instead of the median consumption of fruits and/or vegetables 3 times/day. We also considered alternative aspects of the original dietary factors, including the sodium-to-potassium ratio and sodium density (mg/1,000 kcal). Furthermore, to explore the association between individual HLFs and our outcomes, we stratified individuals by other risk factors such as sex, age, household income, FH of these conditions, and BMI. Our analyses indicated that these various considerations did not substantially alter our results, except for the association between BMI groups and outcomes when stratified by age groups (Supplementary Materials 4-9).

## DISCUSSION

The findings of this study demonstrate a clear inverse association between HLFs and the risk of comorbid hypertension and dyslipidemia, as well as each condition on its own. Notably, among the various individual HLFs, BMI status stood out as a significant factor influencing the risk of both hypertension and dyslipidemia, as well as their comorbidity. Healthy alcohol consumption and adequate intake of fruits and vegetables were associated with a reduced risk of hypertension, while non-smoking was associated with a lower risk of dyslipidemia. Furthermore, as individuals’ overall HLS improved, there was a clear trend of decreasing odds for both hypertension and dyslipidemia, as well as their comorbidity, independent of other covariates.

Our findings are consistent with those of previous studies demonstrating a consistent pattern despite minor variations in HLFs [13,31,32]. These studies have consistently demonstrated a link between adherence to healthier lifestyles and a reduced risk of the conditions under investigation. While BMI has been consistently identified as a significant risk factor, highlighting the critical role of obesity in disease prevention, individual HLFs have shown less agreement than BMI; however, they primarily indicated inverse associations with hypertension and dyslipidemia [13,31-35]. In our study, we noted that favorable lifestyle factors, such as healthy alcohol consumption and adequate fruit and vegetable intake, were inversely associated with hypertension. In contrast, non-smoking was inversely associated with dyslipidemia, and active PA was significantly associated with their comorbidity. To assess the impact of individual HLFs independently of BMI, we performed a stratified analysis by BMI categories. Across all BMI groups, we consistently found inverse associations between individual HLFs and the conditions studied, despite some inconsistencies among individual HLFs concerning hypertension, dyslipidemia, and their comorbidity.

However, the associations between individual HLFs and hypertension have yielded inconclusive results, with the exception of BMI [13,31,32]. Several prospective studies involving middle-aged adults from European and Australian populations have reported varying effect sizes for individual associations among HLFs, while consistently indicating their directions. It is important to recognize that the observed discrepancies in effects could potentially be attributed to variations in confounding variables such as age and sex, sample sizes, levels and methods of assessing exposure to lifestyle factors, definitions of cut-off points, and types and amounts of alcohol consumed [31,32]. Moreover, given that these studies were predominantly conducted in Western countries, differences in genetic backgrounds may also contribute to such discrepancies.

The associations between HLFs and dyslipidemia have shown mixed results in 3 cross-sectional studies, with the exception of obesity [33-35]. Although these analyses did not consider dietary factors, some studies have identified significant associations between smoking, PA, and dyslipidemia. As for alcohol consumption, the relationship with dyslipidemia has not shown a consistent pattern, and variability in individual lipid parameters has been observed across different study populations [33-35]. These complex relationships highlight the heterogeneous impact of alcohol on lipid profiles, which varies based on the type and quantity of alcohol consumed [33-35]. Further research is needed to fully understand the individual and combined effects of HLFs. However, it is prudent to exercise caution with alcohol consumption to prevent binge or heavy drinking, given its well-documented negative impacts on various health aspects, including hemorrhagic stroke, hypertension, and various cancers [36].

When conducting sensitivity analyses using obesity indicators beyond the commonly used BMI for classifying overweight and obese individuals, such as WC and WHtR, our results consistently demonstrated robustness and, in some instances, stronger associations than those observed with BMI. This consistency aligns with the findings of previous studies that reported similar associations between these obesity indicators and chronic diseases [37], with some studies indicating even more pronounced associations [38-40]. Similarly, in our study on the association between PA and hypertension and dyslipidemia, we considered various factors, such as different types of walking, muscle strength, and sedentary time, compared to adherence to the WHO guidelines, which we used in our study. Our results revealed an inverse association with comorbidity, consistent with the findings of previous research [27]. However, we observed that anaerobic exercises, such as muscle strength, exhibited a relevant relationship with hypertension, whereas walking time and sedentary time were associated with dyslipidemia. Further studies are warranted to provide additional evidence on the volume and intensity of PA and its specific impact on health outcomes, as gaps remain in our understanding [41].

While variations in significance and effect sizes may exist for individual factors, our results consistently showed that a higher HLS was associated with lower odds for both the individual and combined conditions. Even after stratifying by BMI, revealing the relevant effects of hypertension and dyslipidemia, and adjusting for BMI as another HLF in the multivariable model, a higher HLS, excluding BMI, was still associated with lower odds of these conditions. From a public health standpoint, it is essential to prioritize comprehensive indicators over individual ones due to the crucial consideration of the interrelations between various HLFs [12-14,23,31,32]. Furthermore, while non-modifiable risk factors such as demographic factors and health-related factors (FH of these conditions and BMI) are significant contributors to the risk of hypertension and dyslipidemia [1], our results demonstrated that modifying HLFs could reduce the risk of these conditions regardless of various covariates [15,42,43]. This highlights the substantial potential for lifestyle interventions to prevent and manage these common health issues and offers valuable insights into public health strategies and clinical practice.

However, it is essential to acknowledge the inherent limitations of our study design. First, due to its cross-sectional nature, our study lacks the temporal dimension necessary to establish causality. Therefore, further prospective studies are required to confirm our findings in the future. Second, a significant portion of our data was based on self-reported information, except for anthropometric measurements, BP assessments, and lipid profiles. Consequently, the potential for underreporting certain behaviors due to recall bias may have led to an underestimation of the observed effects, despite our efforts to standardize processes and minimize bias to increase accuracy. Despite considering numerous potential confounding factors, including self-reported FH of these conditions, it is crucial to acknowledge that our study has limitations related to the absence of genetic information. We cannot definitively rule out the possibility that the observed associations were influenced by residual or unmeasured confounders, including genetic factors, which were not within the scope of our investigation. Nevertheless, our study has several strengths. We utilized nationally representative data and included additional information, such as anthropometric measurements (WC and WHtR), dietary sodium intake, and various forms of PA and FH. Notably, to the best of our knowledge, this study is the first comprehensive investigation of the associations between individual and combined lifestyle scores and hypertension, dyslipidemia, and their comorbidity in Korean adults.

In conclusion, our study provides evidence that HLFs are associated not only with the individual risks of hypertension and dyslipidemia but also with the comorbidity of these conditions. These associations remained consistent regardless of the covariates. Moreover, our findings underscore the importance of incorporating multidimensional HLFs to improve our understanding of primary prevention and management strategies for chronic diseases such as hypertension and dyslipidemia, whether they occur independently or coexist as comorbid conditions. Our results emphasize the need for further epidemiological investigations to comprehensively understand the joint associations between HLFs and chronic diseases and their comorbidities.

## Figures and Tables

**Figure f2-epih-46-e2024049:**
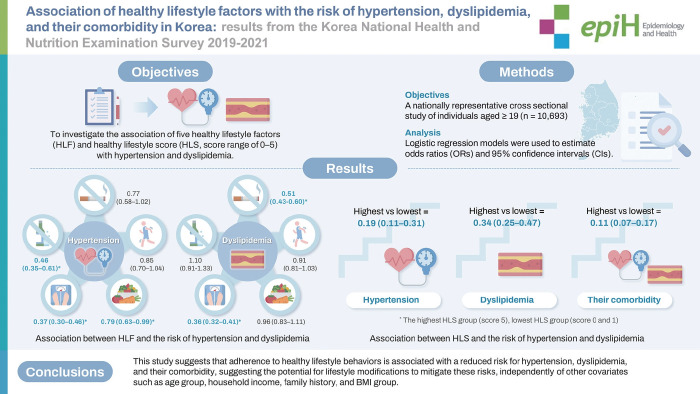


**Table 1.** Description and scoring criteria for five lifestyle factors

Lifestyle factor	Classification	Point	Description
Smoking	Smoking	0	Current smoker and ≥100 cigarettes in their lifetime
	Non-smoking	1	Never smoker, former smoker, or <100 cigarettes in their lifetime
Alcohol drinking	Unhealthy consumption	0	>2 drink/day for male and >1 drink/day for female
	Low consumption	1	No drinking or ≤2 drink/day for male and ≤1 drink/day for female
BMI status (kg/m^2^)	Obesity	0	≥25
	Non-obesity	1	<25
Fruit and vegetable consumption	Unhealthy	0	Consumption of fruit and vegetables per day <median (3 times)
	Healthy	1	Consumption of fruit and vegetables per day ≥median (3 times)
PA	Unhealthy	0	Did not meet WHO guidelines on PA: <150 min of moderate-intensity aerobic PA or at least 75-150 min of vigorous-intensity aerobic PA; or an equivalent combination of moderate- and vigorous-intensity PA throughout the week
	Healthy	1	Meet WHO guidelines on PA: at least 150 min of moderate-intensity aerobic PA or at least 75-150 min of vigorous-intensity aerobic PA; or an equivalent combination of moderate- and vigorous-intensity PA throughout the week
Range			0 (unhealthy) to 5 (healthy)

BMI, body mass index; PA, physical activity; WHO, World Health Organization.

**Table 2.** Population characteristics according to healthy lifestyle score in a nationally representative sample of Korean adults, KNHANES 2019-2021 (n=10,693)

Characteristics	Total	Healthy lifestyle score					
		0-1 (lowest)	2	3	4	5 (highest)	p-value
Toatl, n (%)^1^	10,693	564 (6.41)	1,486 (15.5)	3,439 (32.9)	3,645 (31.7)	1,559 (13.5)	0.002
Male	50.9	81.8	68.3	51.6	42.0	35.4	<0.001
Age (yr)	42.5±0.31	41.4±0.55	43.4±0.39	44.5±0.37	47.1±0.35	48.5±0.48	<0.001
19-39	38.1	46.6	42.7	41.8	34.5	28.4	<0.001
40-59	41.5	45.3	44.2	38.8	40.2	46.6	
60-79	20.3	8.1	13.1	19.4	25.3	25.1	
Education level							
≤Elementary school	8.4	5.5	6.7	8.7	10.2	7.1	<0.001
Middle school	6.7	7.0	6.6	6.8	6.4	7.1	
High school	37.2	47.7	37.5	38.4	35.1	33.9	
≥ College	47.7	39.9	49.3	46.2	48.3	51.9	
Household income							
Low	11.2	12.2	10.8	12.0	11.4	9.0	0.003
Low-middle	22.3	22.4	22.7	22.6	22.9	19.9	
Middle-high	30.3	35.6	31.7	30.1	29.0	29.5	
High	36.1	29.8	34.7	35.3	36.7	41.6	
Marital status, married	72.2	65.4	71.3	68.7	74.5	79.3	<0.001
Healthy lifestyle factors							
Non-smoking	81.0	16.7	56.3	82.7	96.0	100	-
Low alcohol consumption	83.6	25.6	62.5	86.5	95.6	100	-
Healthy physical activity	46.9	11.3	25.6	33.8	55.5	100	-
Healthy BMI status	63.6	23.7	33.8	54.5	80.1	100	-
Favorable fruit and vegetable intakes	54.4	6.8	21.7	42.4	72.8	100	-
Hypertension	23.7	32.3	30.1	23.3	21.6	17.8	<0.001
Dyslipidemia	39.6	52.3	46.9	40.4	35.9	31.6	<0.001
Blood pressure (mmHg)							
SBP	117±0.21	123±0.70	120±0.43	117±0.32	116±0.32	114±0.45	<0.001
DBP	74.4±0.13	81.2±0.54	78.8±0.31	75.6±0.22	74.0±0.19	73.2±0.26	<0.001
Lipid profile (mg/dL)							
TC	189±0.39	198±2.01	196±1.09	193±0.78	191±0.72	193±1.10	<0.001
TG	126±1.06	192±6.46	162±3.78	129±1.97	111±1.54	103±2.25	<0.001
LDL-C	113±0.34	116±1.86	117±1.00	117±0.68	116±0.64	116±0.97	0.870
HDL-C	52.2±0.14	48.8±0.60	50.4±0.41	51.7±0.27	53.6±0.26	55.9±0.36	<0.001
BMI (kg/m^2^)	23.3±0.05	26.6±0.17	26.2±0.13	24.6±0.09	23.0±0.06	21.9±0.07	<0.001
Total energy intake (kcal/day)	1,877±10.0	2,278±55.6	2,082±30.8	1,913±19.7	1,888±16.8	1,859±25.1	<0.001

All values accounted for the complex sampling design effect of the national surveys using PROC SURVEY procedure, and values are presented as mean±standard error or %. KNHANES, Korea National Health and Nutrition Examination Survey; BMI, body mass index; SBP, systolic blood pressure; DBP, diastolic blood pressure; TC, total cholesterol; TG, triglyceride; LDL-C, low-density lipoprotein cholesterol; HDL-C, high-density lipoprotein.cholesterol

1 The unweighted sample size for each subgroup.

**Table 3.** Association between individual components of healthy lifestyle factors and the risk of hypertension and/or dyslipidemia using multinomial logistic analysis (n=10,693)^1^

Variables	Hypertension alone	Dyslipidemia alone	Hypertension and dyslipidemia
Total (n)	1,127	2,616	1,899
Non-smoking			
Model 1	0.77 (0.60, 0.98)	0.67 (0.57, 0.78)	0.64 (0.52, 0.79)
Model 2	0.75 (0.56, 1.01)	0.66 (0.56, 0.78)	0.61 (0.45, 0.82)
Model 3	0.77 (0.58, 1.02)	0.51 (0.43, 0.60)	0.51 (0.38, 0.68)
Low alcohol consumption			
Model 1	0.46 (0.36, 0.59)	1.03 (0.87, 1.23)	0.60 (0.50, 0.72)
Model 2	0.43 (0.32, 0.57)	1.02 (0.85, 1.22)	0.54 (0.42, 0.70)
Model 3	0.46 (0.35, 0.61)	1.10 (0.91, 1.33)	0.57 (0.43, 0.74)
Healthy body mass index status			
Model 1	0.33 (0.28, 0.39)	0.40 (0.36, 0.46)	0.18 (0.15, 0.21)
Model 2	0.40 (0.33, 0.49)	0.41 (0.36, 0.46)	0.22 (0.18, 0.27)
Model 3	0.37 (0.30, 0.46)	0.36 (0.32, 0.41)	0.20 (0.16, 0.24)
Healthy fruit and vegetable consumption			
Model 1	0.78 (0.64, 0.94)	0.93 (0.81, 1.05)	0.82 (0.69, 0.98)
Model 2	0.72 (0.57, 0.90)	0.93 (0.81, 1.06)	0.81 (0.63, 1.02)
Model 3	0.79 (0.63, 0.99)	0.96 (0.83, 1.11)	0.91 (0.70, 1.16)
Healthy physical activity			
Model 1	0.88 (0.75, 1.03)	0.92 (0.82, 1.03)	0.77 (0.66, 0.89)
Model 2	0.83 (0.68, 1.01)	0.88 (0.78, 1.00)	0.67 (0.55, 0.81)
Model 3	0.85 (0.70, 1.04)	0.91 (0.81, 1.03)	0.69 (0.56, 0.85)

Values are presented as odds ratio (95% confidence interval).

1 Model 1: adjusted for age (years) and sex; Model 2: adjusted for age (years), sex, education status (≥12 years of school), household income status (≥3 times/wk and ≥30 min/session), marital status, energy intake (kcal/day), diagnosis of hypertension and/or dyslipidemia by physician, and family history of hypertension and/or dyslipidemia; Model 3 was adjusted for the covariates in model 2 and includes the other lifestyle factors.

**Table 4.** Association between healthy lifestyle score and the risk of hypertension and/or dyslipidemia using multinomial logistic analysis (n=10,693)

Healthy lifestyle score	Hypertension alone (n=1,127)		Dyslipidemia alone (n=2,616)		Hypertension and dyslipidemia (n=1,899)	
	Age and sex-adjusted	Multivariable^1^	Age and sex-adjusted	Multivariable^1^	Age and sex-adjusted	Multivariable^1^
0-1 (lowest)	1.00 (reference)	1.00 (reference)	1.00 (reference)	1.00 (reference)	1.00 (reference)	1.00 (reference)
2	0.60 (0.40, 0.92)	0.60 (0.37, 0.96)	0.70 (0.52, 0.95)	0.72 (0.52, 0.98)	0.65 (0.47, 0.91)	0.63 (0.42, 0.96)
3	0.35 (0.23, 0.52)	0.34 (0.22, 0.54)	0.58 (0.44, 0.75)	0.58 (0.44, 0.77)	0.32 (0.24, 0.44)	0.30 (0.20, 0.44)
4	0.23 (0.16, 0.34)	0.23 (0.15, 0.36)	0.42 (0.32, 0.55)	0.41 (0.31, 0.55)	0.19 (0.14, 0.27)	0.18 (0.12, 0.27)
5 (highest)	0.19 (0.12, 0.30)	0.19 (0.11, 0.31)	0.36 (0.26, 0.48)	0.34 (0.25, 0.47)	0.11 (0.08, 0.16)	0.11 (0.07, 0.17)
p for trend	<0.001	<0.001	<0.001	<0.001	<0.001	<0.001
Every 1-point increment	0.66 (0.60, 0.72)	0.65 (0.59, 0.72)	0.78 (0.73, 0.82)	0.76 (0.72, 0.81)	0.57 (0.53, 0.61)	0.56 (0.51, 0.62)

Values are presented as odds ratio (95% confidence interval).

1 The multivariable model was adjusted for age (years), sex, education status (≥12 years of school), household income status (≥3 times/wk and ≥30 min/session), marital status, energy intake (kcal/day), diagnosis of hypertension and/or dyslipidemia by a physician, and family history of hypertension and/or dyslipidemia.

## Supplementary materials

Supplementary Material 1. Selection of participants from KNHANES 2019–2021

Supplementary Material 2. Correlation coefficients between healthy lifestyle factors and healthy lifestyle scores (n=10,693)

Supplementary Material 3. Sensitivity analyses of the association between healthy lifestyle scores and the risk of comorbidity status of hypertension and dyslipidemia using multinomial logistic regression (n=10,693)

Supplementary Material 4. Association between individual components of healthy lifestyle factors and the risk of hypertension and dyslipidemia by sex (n=10,693)

Supplementary Material 5. Association between individual components of healthy lifestyle factors and the risk of hypertension and dyslipidemia by age group (n=10,693)

Supplementary Material 6. Association between individual components of healthy lifestyle factors and risk of hypertension and dyslipidemia by household income (n=10,693)

Supplementary Material 7. Association between individual components of healthy lifestyle factor and risk of hypertension and dyslipidemia by family history (n=10,693)

Supplementary Material 8. Association between the individual components of healthy lifestyle factors and the risk of hypertension and dyslipidemia by BMI group (n=10,693)

Supplementary Material 9. Association between the individual components of healthy lifestyle factors and the risk of hypertension and dyslipidemia by prior physician diagnoses of hypertension and/or dyslipidemia (n=10,693)
