# Supplementary material for: Association of healthy lifestyle factors with the risk of hypertension, dyslipidemia, and their comorbidity in Korea: results from the Korea National Health and Nutrition Examination Survey 2019-2021
Source: Epidemiol Health. 2024 May 1;46:e2024049. doi: 10.4178/epih.e2024049 (PMC11417455; doi:10.4178/epih.e2024049)
Supplement: Supplementary Material 1. — Selection of participants from KNHANES 2019–2021 [file epih-46-e2024049-Supplementary-1.docx]

**
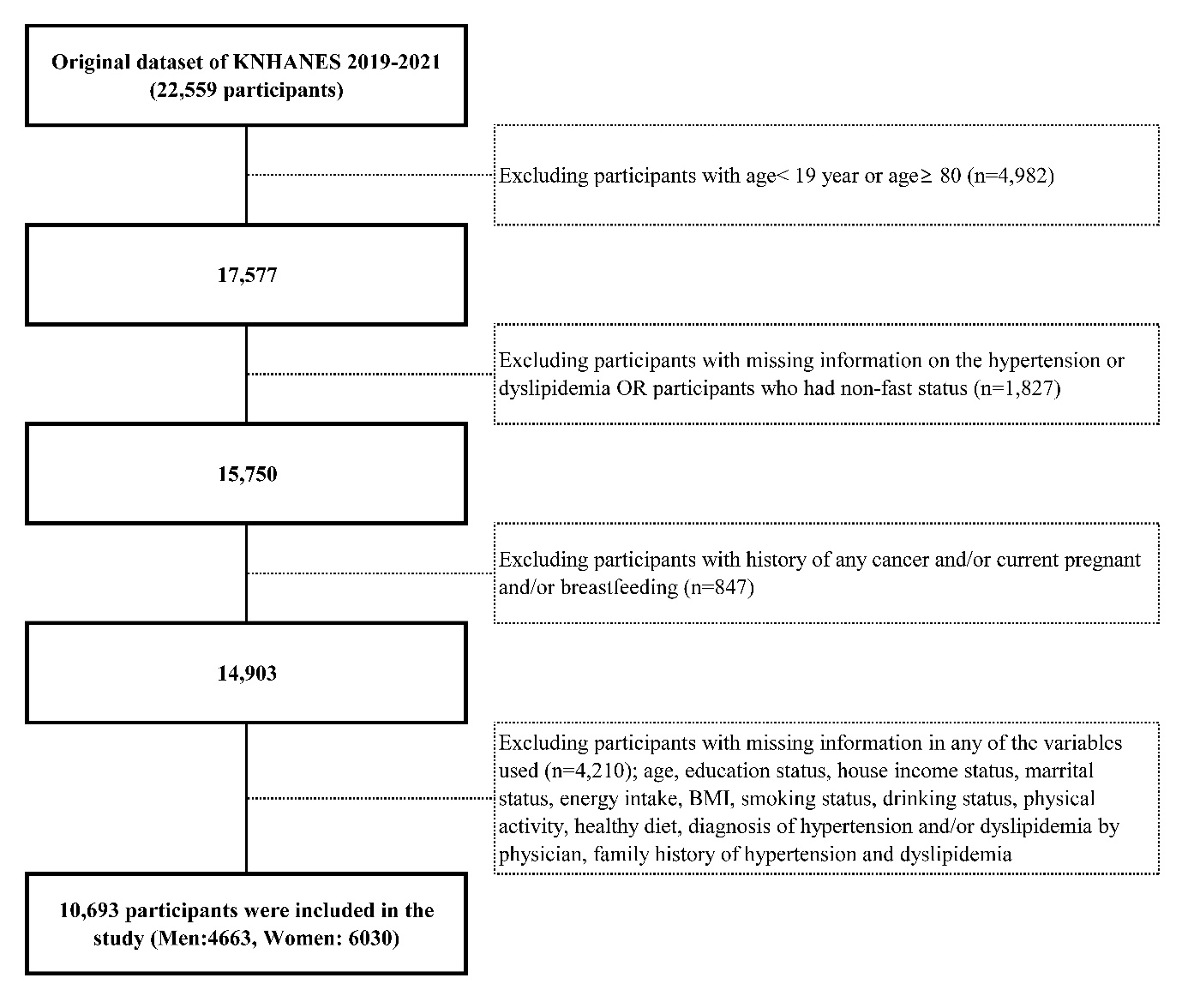
**

**Supplemental Material 1**. Selection of participants from KNHANES 2019–2021.

Abbreviations: KNHANES, Korea National Health and Nutrition Examination Survey.
