# Supplementary material for: Association of healthy lifestyle factors with the risk of hypertension, dyslipidemia, and their comorbidity in Korea: results from the Korea National Health and Nutrition Examination Survey 2019-2021
Source: Epidemiol Health. 2024 May 1;46:e2024049. doi: 10.4178/epih.e2024049 (PMC11417455; doi:10.4178/epih.e2024049)
Supplement: Supplementary Material 2. — Correlation coefficients between healthy lifestyle factors and healthy lifestyle scores (n=10,693) [file epih-46-e2024049-Supplementary-2.docx]

**Supplemental Material 2.** Correlation coefficients between healthy lifestyle factors and healthy lifestyle scores (n=10,693)

| **Lifestyle factor** | Healthy lifestyle scores | Non-smoking | Low alcohol consumption | Healthy physical activity | Healthy fruit and vegetable intakes | Non-obesity |
| --- | --- | --- | --- | --- | --- | --- |
| Healthy lifestyle scores |  | 0.42^***^ | 0.40^***^ | 0.49^***^ | 0.54^***^ | 0.53^***^ |
| Non-smoking | 0.42^***^ |  | 0.19^***^ | 0.002 | 0.10^***^ | 0.05^***^ |
| Low alcohol consumption | 0.40^***^ | 0.19^***^ |  | 0.03^***^ | 0.08^***^ | 0.008 |
| Healthy physical activity | 0.49^***^ | 0.002 | 0.03^***^ |  | 0.03^***^ | 0.008 |
| Healthy fruit and vegetable intakes | 0.54^***^ | 0.10*** | 0.08^***^ | 0.03^***^ |  | 0.07^***^ |
| Non-obesity | 0.53^***^ | 0.05^***^ | 0.008 | 0.008 | 0.07^***^ |  |

Spearman’s correlation coefficients and *p* value between individual lifestyle factors and healthy lifestyle scores.

Partial age (continuous) and sex.

^*^ *p* <0.05, ^**^ *p* <0.01, ^***^ *p* <0.001.
