# Supplementary material for: Association of healthy lifestyle factors with the risk of hypertension, dyslipidemia, and their comorbidity in Korea: results from the Korea National Health and Nutrition Examination Survey 2019-2021
Source: Epidemiol Health. 2024 May 1;46:e2024049. doi: 10.4178/epih.e2024049 (PMC11417455; doi:10.4178/epih.e2024049)
Supplement: Supplementary Material 3. — Sensitivity analyses of the association between healthy lifestyle scores and the risk of comorbidity status of hypertension and dyslipidemia using multinomial logistic regression (n=10,693) [file epih-46-e2024049-Supplementary-3.docx]

**Supplemental Material 3.** Sensitivity analyses of the association between healthy lifestyle scores and the risk of comorbidity status of hypertension and dyslipidemia using multinomial logistic regression (n=10,693)

| **Healthy lifestyle score** | **Hypertension alone**  **OR (95% CI)** | **Dyslipidemia alone**  **OR (95% CI)** | **Hypertension**  **and dyslipidemia**  **OR (95% CI)** |
| --- | --- | --- | --- |
| ***N*** | 1,127 | 2,616 | 1,899 |
| **Obesity factor** |  |  |  |
| By replacing the BMI with waist circumference (men ≥90 cm, women ≥85cm) | **0.34 (0.28–0.42)** | **0.35 (0.30–0.40)** | **0.19 (0.16–0.23)** |
| By replacing the BMI with waist-to-height ratio (≥0.5) | **0.36 (0.30–0.45)** | **0.33 (0.29–0.37)** | **0.18 (0.14–0.22)** |
|  |  |  |  |
| **Physical activity factor** |  |  |  |
| By replacing original factor with walking time (≥3 times/week and ≥30 min/week) | 0.84 (0.68–1.04) | **0.85 (0.74–0.97)** | **0.72 (0.59–0.88)** |
| By replacing original factor with muscle exercise (≥2 times/week) | **0.75 (0.59–0.97)** | 0.92 (0.79–1.05) | **0.63 (0.50–0.79)** |
| By replacing original factor with seating time (≥8 hours/day) | 0.94 (0.76–1.18) | **0.88 (0.78–1.00)** | 0.86 (0.71–1.05) |
|  |  |  |  |
| **Dietary factor** |  |  |  |
| By using five times per day consumption of fruits and/or vegetables | 0.77 (0.53–1.12) | **0.68 (0.52–0.91)** | 0.76 (0.52–1.10) |
| By using four times per day consumption of fruits and/or vegetables | **0.63 (0.50–0.80)** | **0.85 (0.72–0.99)** | 0.85 (0.68–1.08) |
| By replacing the FV factor with consumption of fruit (median) | 1.01 (0.81–1.25) | 1.10 (0.96–1.25) | 0.95 (0.76–1.19) |
| By replacing the FV factor with consumption of vegetables (median) | 0.83 (0.59–1.18) | 0.99 (0.82–1.20) | 1.01 (0.71–1.42) |
| By replacing the FV factor with sodium density (median) | **0.81 (0.67-0.99)** | 0.93 (0.82-1.05) | 0.90 (0.75-1.09) |
| By replacing the FV factor with Sodium: Potassium (median) | 1.02 (0.84–1.23) | 1.05 (0.93–1.19) | 0.97 (0.80–1.18) |
|  |  |  |  |

Abbreviations: OR, odds ratio; CI, confidence interval; FV, fruits and/or vegetables.

The multivariable model was adjusted for age, sex, education level, household income status, marital status, energy intake, diagnosis of hypertension and/or dyslipidemia by physicians, family history of hypertension and/or dyslipidemia, and other lifestyle factor
