# Supplementary material for: Association of healthy lifestyle factors with the risk of hypertension, dyslipidemia, and their comorbidity in Korea: results from the Korea National Health and Nutrition Examination Survey 2019-2021
Source: Epidemiol Health. 2024 May 1;46:e2024049. doi: 10.4178/epih.e2024049 (PMC11417455; doi:10.4178/epih.e2024049)
Supplement: Supplementary Material 4. — Association between individual components of healthy lifestyle factors and the risk of hypertension and dyslipidemia by sex (n=10,693) [file epih-46-e2024049-Supplementary-4.docx]

**Supplemental Material 4.** Association between individual components of healthy lifestyle factors and the risk of hypertension and dyslipidemia by sex (n=10,693)

| **Variables** | **Hypertension alone OR (95% CI)** | **Dyslipidemia alone OR (95% CI)** | **Hypertension and dyslipidemia**  **OR (95% CI)** | ***P* for interaction** |
| --- | --- | --- | --- | --- |
| **Non-smoking** |  |  |  |  |
| Men | 0.85 (0.62–1.16) | **0.60 (0.50–0.73)** | **0.68 (0.50–0.91)** | 0.4784 |
| Women | 1.05 (0.48–2.27) | 0.85 (0.58–1.25) | 0.56 (0.25–1.22) |  |
| **Low alcohol consumption** |  |  |  |  |
| Men | 0.48 (0.35–0.66) | 1.12 (0.89–1.41) | 0.57 (0.43–0.76) | 0.6308 |
| Women | 0.36 (0.22–0.61) | 1.05 (0.77–1.43) | 0.61 (0.34–1.09) |  |
| **Non-obesity** |  |  |  |  |
| Men | **0.45 (0.33–0.62)** | **0.41 (0.35–0.48)** | **0.21 (0.16–0.27)** | 0.0902 |
| Women | **0.38 (0.28–0.51)** | **0.43 (0.35–0.52)** | **0.26 (0.20–0.35)** |  |
| **Healthy fruit and vegetables status** |  |  |  |  |
| Men | 0.78 (0.56–1.10) | 1.02 (0.83–1.25) | 0.89 (0.65–1.22) | 0.1127 |
| Women | 0.88 (0.64–1.20) | 0.93 (0.76–1.15) | 1.04 (0.71–1.52) |  |
| **Healthy physical activity** |  |  |  |  |
| Men | 0.84 (0.63–1.12) | 0.88 (0.74–1.05) | **0.58 (0.44–0.76)** | 0.2461 |
| Women | 0.83 (0.62–1.09) | 0.87 (0.73–1.04) | 0.84 (0.61–1.16) |  |
|  |  |  |  |  |

Abbreviations: OR, odds ratio; CI, confidence interval.

The multivariable model was adjusted for age, education level, household income status, marital status, energy intake, diagnosis of hypertension and/or dyslipidemia by physicians, family history of hypertension and/or dyslipidemia, and other lifestyle factors.
