# Supplementary material for: Association of healthy lifestyle factors with the risk of hypertension, dyslipidemia, and their comorbidity in Korea: results from the Korea National Health and Nutrition Examination Survey 2019-2021
Source: Epidemiol Health. 2024 May 1;46:e2024049. doi: 10.4178/epih.e2024049 (PMC11417455; doi:10.4178/epih.e2024049)
Supplement: Supplementary Material 5. — Association between individual components of healthy lifestyle factors and the risk of hypertension and dyslipidemia by age group (n=10,693) [file epih-46-e2024049-Supplementary-5.docx]

**Supplemental Material 5.** Association between individual components of healthy lifestyle factors and the risk of hypertension and dyslipidemia by age group (n=10,693)

| **Variables** | **Hypertension alone**  **OR (95% CI)** | **Dyslipidemia alone**  **OR (95% CI)** | **Hypertension and dyslipidemia**  **OR (95% CI)** | ***P* for interaction** |
| --- | --- | --- | --- | --- |
| **Non-smoking** |  |  |  |  |
| <50 | 0.80 (0.54–1.19) | **0.53 (0.43–0.66)** | **0.44 (0.29–0.65)** | 0.3199 |
| ≥50 | 0.85 (0.56–1.31) | **0.50 (0.38–0.65)** | **0.67 (0.45–0.99)** |  |
| **Low alcohol consumption** |  |  |  |  |
| <50 | **0.39 (0.27–0.55)** | 1.18 (0.93–1.48) | **0.68 (0.47–0.99)** | 0.4783 |
| ≥50 | **0.58 (0.39–0.88)** | 0.98 (0.72–1.34) | **0.56 (0.38–0.83)** |  |
| **Non-obesity** |  |  |  |  |
| <50 | **0.37 (0.27–0.52)** | **0.28 (0.24–0.34)** | **0.11 (0.08–0.15)** | **<0.0001** |
| ≥50 | **0.52 (0.40–0.68)** | **0.61 (0.50–0.75)** | **0.35 (0.27–0.45)** |  |
| **Healthy fruit and vegetables status** |  |  |  |  |
| <50 | 0.80 (0.56–1.14) | 0.99 (0.83–1.18) | 1.02 (0.71–1.46) | 0.3145 |
| ≥50 | 0.74 (0.54–1.01) | 0.92 (0.74–1.16) | 0.80 (0.58–1.10) |  |
| **Healthy physical activity** |  |  |  |  |
| <50 | 0.76 (0.55–1.04) | 0.88 (0.75–1.04) | **0.61 (0.45–0.84)** | 0.3685 |
| ≥50 | 0.98 (0.76–1.27) | 0.95 (0.78–1.15) | 0.79 (0.59–1.05) |  |
|  |  |  |  |  |

Abbreviations: OR, odds ratio; CI, confidence interval.

The multivariable model was adjusted for age, sex, education level, household income status, marital status, energy intake, diagnosis of hypertension and/or dyslipidemia by physicians, family history of hypertension and/or dyslipidemia, and other lifestyle factors.
