# Supplementary material for: Association of healthy lifestyle factors with the risk of hypertension, dyslipidemia, and their comorbidity in Korea: results from the Korea National Health and Nutrition Examination Survey 2019-2021
Source: Epidemiol Health. 2024 May 1;46:e2024049. doi: 10.4178/epih.e2024049 (PMC11417455; doi:10.4178/epih.e2024049)
Supplement: Supplementary Material 6. — Association between individual components of healthy lifestyle factors and risk of hypertension and dyslipidemia by household income (n=10,693) [file epih-46-e2024049-Supplementary-6.docx]

**Supplemental Material 6.** Association between individual components of healthy lifestyle factors and risk of hypertension and dyslipidemia by household income (n=10,693)

| **Variables** | **Hypertension alone**  **OR (95% CI)** | **Dyslipidemia alone**  **OR (95% CI)** | **Hypertension and dyslipidemia**  **OR (95% CI)** | ***P* for interaction** |
| --- | --- | --- | --- | --- |
| **Non-smoking** |  |  |  |  |
| Lower income | 0.78 (0.50–1.22) | **0.59 (0.45–0.79)** | **0.54 (0.36–0.80)** | 0.7716 |
| Higher income | 0.76 (0.52–1.11) | **0.47 (0.38–0.57)** | **0.49 (0.33–0.72)** |  |
| **Low alcohol consumption** |  |  |  |  |
| Lower income | **0.53 (0.33–0.84)** | 1.11 (0.79–1.55) | **0.61 (0.39–0.95)** | 0.8316 |
| Higher income | **0.43 (0.31–0.60)** | 1.11 (0.89–1.38) | **0.56 (0.40–0.77)** |  |
| **Non-obesity** |  |  |  |  |
| Lower income | **0.42 (0.30–0.58)** | **0.43 (0.35–0.54)** | **0.21 (0.15–0.28)** | 0.4119 |
| Higher income | **0.35 (0.27–0.45)** | **0.33 (0.28–0.39)** | **0.20 (0.15–0.25)** |  |
| **Healthy fruit and vegetables status** |  |  |  |  |
| Lower income | 0.75 (0.52–1.09) | 0.93 (0.74–1.17) | 0.92 (0.61–1.40) | 0.7131 |
| Higher income | 0.81 (0.60–1.11) | 0.96 (0.81–1.15) | 0.88 (0.66–1.17) |  |
| **Healthy physical activity** |  |  |  |  |
| Lower income | 0.93 (0.67–1.31) | 0.88 (0.71–1.09) | 0.75 (0.53–1.07) | 0.8101 |
| Higher income | 0.81 (0.63–1.04) | 0.92 (0.79–1.07) | **0.67 (0.52–0.85)** |  |
|  |  |  |  |  |

Abbreviations: OR, odds ratio; CI, confidence interval.

The multivariable model was adjusted for age, sex, education level, household income status, marital status, energy intake, diagnosis of hypertension and/or dyslipidemia by physicians, family history of hypertension and/or dyslipidemia, and other lifestyle factors.
