# Supplementary material for: Association of healthy lifestyle factors with the risk of hypertension, dyslipidemia, and their comorbidity in Korea: results from the Korea National Health and Nutrition Examination Survey 2019-2021
Source: Epidemiol Health. 2024 May 1;46:e2024049. doi: 10.4178/epih.e2024049 (PMC11417455; doi:10.4178/epih.e2024049)
Supplement: Supplementary Material 7. — Association between individual components of healthy lifestyle factor and risk of hypertension and dyslipidemia by family history (n=10,693) [file epih-46-e2024049-Supplementary-7.docx]

**Supplemental Material 7.** Association between individual components of healthy lifestyle factor and risk of hypertension and dyslipidemia by family history (n=10,693)

| **Variables** | **Hypertension alone**  **OR (95% CI)** | **Dyslipidemia alone**  **OR (95% CI)** | **Hypertension and dyslipidemia**  **OR (95% CI)** | ***P* for interaction** |
| --- | --- | --- | --- | --- |
| **Non-smoking** |  |  |  |  |
| With family history | 0.93 (0.61–1.42) | **0.60 (0.46–0.79)** | 0.66 (0.43–1.01) | 0.648 |
| Without family history | 0.85 (0.57–1.26) | **0.68 (0.54–0.84)** | 0.68 (0.45–1.02) |  |
| **Low alcohol consumption** |  |  |  |  |
| With family history | **0.53 (0.36–0.77)** | 1.19 (0.91–1.56) | 0.74 (0.51–1.09) | 0.3536 |
| Without family history | **0.40 (0.28–0.57)** | 1.11 (0.85–1.44) | **0.47 (0.33–0.67)** |  |
| **Non-obesity** |  |  |  |  |
| With family history | **0.45 (0.33–0.60)** | **0.41 (0.33–0.50)** | **0.23 (0.18–0.30)** | 0.8861 |
| Without family history | **0.35 (0.26–0.46)** | **0.40 (0.34–0.46)** | **0.20 (0.15–0.27)** |  |
| **Healthy fruit and vegetables status** |  |  |  |  |
| With family history | 0.90 (0.65–1.25) | 0.89 (0.71–1.10) | 1.08 (0.76–1.54) | 0.1407 |
| Without family history | **0.67 (0.48–0.94)** | 1.07 (0.89–1.28) | 0.78 (0.56–1.08) |  |
| **Healthy physical activity** |  |  |  |  |
| With family history | **0.68 (0.51–0.90)** | **0.74 (0.61–0.89)** | **0.58 (0.43–0.78)** | 0.1342 |
| Without family history | 1.01 (0.76–1.35) | 1.01 (0.86–1.19) | **0.76 (0.59–0.99)** |  |
|  |  |  |  |  |

Abbreviations: OR, odds ratio; CI, confidence interval.

The multivariable model was adjusted for age, sex, education level, household income status, marital status, energy intake, diagnosis of hypertension and/or dyslipidemia by physicians, and other lifestyle factors.
