# Supplementary material for: Association of healthy lifestyle factors with the risk of hypertension, dyslipidemia, and their comorbidity in Korea: results from the Korea National Health and Nutrition Examination Survey 2019-2021
Source: Epidemiol Health. 2024 May 1;46:e2024049. doi: 10.4178/epih.e2024049 (PMC11417455; doi:10.4178/epih.e2024049)
Supplement: Supplementary Material 8. — Association between the individual components of healthy lifestyle factors and the risk of hypertension and dyslipidemia by BMI group (n=10,693) [file epih-46-e2024049-Supplementary-8.docx]

**Supplemental Material 8.** Association between the individual components of healthy lifestyle factors and the risk of hypertension and dyslipidemia by BMI group (n=10,693)

| **Variables** | **Hypertension alone**  **OR (95% CI)** | **Dyslipidemia alone**  **OR (95% CI)** | **Hypertension and dyslipidemia**  **OR (95% CI)** | ***P* for interaction** |
| --- | --- | --- | --- | --- |
| **Non-smoking** |  |  |  |  |
| <25 kg/m^2^ | 0.70 (0.49–1.01) | **0.50 (0.40–0.63)** | **0.52 (0.34–0.81)** | 0.5216 |
| ≥25 kg/m^2^ | 0.88 (0.57–1.37) | **0.53 (0.41–0.68)** | **0.53 (0.36–0.77)** |  |
| **Low alcohol consumption** |  |  |  |  |
| <25 kg/m^2^ | **0.43 (0.30–0.61)** | 1.00 (0.79–1.28) | **0.49 (0.34–0.71)** | 0.4871 |
| ≥25 kg/m^2^ | **0.50 (0.34–0.73)** | 1.23 (0.94–1.61) | **0.64 (0.44–0.93)** |  |
| **Healthy fruit and vegetables status** |  |  |  |  |
| <25 kg/m^2^ | 0.78 (0.57–1.06) | 0.94 (0.79–1.12) | 0.88 (0.63–1.25) | 0.5804 |
| ≥25 kg/m^2^ | 0.83 (0.58–1.16) | 1.01 (0.79–1.28) | 0.96 (0.68–1.35) |  |
| **Healthy physical activity** |  |  |  |  |
| <25 kg/m^2^ | 0.89 (0.69–1.15) | 0.98 (0.83–1.15) | 0.78 (0.58–1.02) | 0.6824 |
| ≥25 kg/m^2^ | 0.77 (0.56–1.05) | **0.79 (0.65–0.97)** | **0.58 (0.43–0.79)** |  |
|  |  |  |  |  |

Abbreviations: OR, odds ratio; CI, confidence interval.

The multivariable model was adjusted for age, sex, education level, household income status, marital status, energy intake, diagnosis of hypertension and/or dyslipidemia by physicians, family history of hypertension and/or dyslipidemia, and other lifestyle factors.
