# Supplementary material for: Association of healthy lifestyle factors with the risk of hypertension, dyslipidemia, and their comorbidity in Korea: results from the Korea National Health and Nutrition Examination Survey 2019-2021
Source: Epidemiol Health. 2024 May 1;46:e2024049. doi: 10.4178/epih.e2024049 (PMC11417455; doi:10.4178/epih.e2024049)
Supplement: Supplementary Material 9. — Association between the individual components of healthy lifestyle factors and the risk of hypertension and dyslipidemia by prior physician diagnoses of hypertension and/or dyslipidemia (n=10,693) [file epih-46-e2024049-Supplementary-9.docx]

**Supplemental Material 9.** Association between the individual components of healthy lifestyle factors and the risk of hypertension and dyslipidemia by prior physician diagnoses of hypertension and/or dyslipidemia (n=10,693)

| **Variables** | **Hypertension alone**  **OR (95% CI)** | **Dyslipidemia alone**  **OR (95% CI)** | **Hypertension and dyslipidemia**  **OR (95% CI)** |
| --- | --- | --- | --- |
| **With prior physician diagnoses** | 712 | 766 | 1,557 |
| **Without prior physician diagnoses** | 415 | 1,850 | 342 |
|  |  |  |  |
| **Non-smoking** |  |  |  |
| With prior physician diagnoses | 0.79 (0.58–1.08) | **0.61 (0.47–0.81)** | **0.58 (0.45–0.76)** |
| Without prior physician diagnoses | **0.72 (0.53–0.98)** | **0.50 (0.42–0.60)** | **0.44 (0.30–0.64)** |
| **Low alcohol consumption** |  |  |  |
| With prior physician diagnoses | **0.56 (0.41–0.77)** | 1.10 (0.81–1.52) | **0.68 (0.53–0.86)** |
| Without prior physician diagnoses | **0.43 (0.32–0.58)** | 1.11 (0.91–1.35) | **0.59 (0.43–0.81)** |
| **Non-obesity** |  |  |  |
| With prior physician diagnoses | **0.28 (0.23–0.35)** | **0.46 (0.37–0.56)** | **0.18 (0.15–0.21)** |
| Without prior physician diagnoses | **0.40 (0.31–0.51)** | **0.35 (0.31–0.40)** | **0.17 (0.13–0.23)** |
| **Healthy fruit and vegetables status** |  |  |  |
| With prior physician diagnoses | 0.98 (0.76–1.27) | 0.93 (0.75–1.18) | 0.93 (0.74–1.15) |
| Without prior physician diagnoses | **0.78 (0.58–1.04)** | 0.95 (0.82–1.11) | 0.86 (0.63–1.19) |
| **Healthy physical activity** |  |  |  |
| With prior physician diagnoses | 0.95 (0.77–1.18) | 1.09 (0.88–1.33) | **0.81 (0.67–0.97)** |
| Without prior physician diagnoses | 0.85 (0.66–1.10) | 0.90 (0.79–1.03) | **0.76 (0.59–0.98)** |
|  |  |  |  |

Abbreviations: OR, odds ratio; CI, confidence interval.

The multivariable model was adjusted for age, sex, education level, household income status, marital status, energy intake, diagnosis of hypertension and/or dyslipidemia by physicians, family history of hypertension and/or dyslipidemia, and other lifestyle factors.
